# The Improvement of Emotion and Attention Regulation after a 6-Week Training of Focused Meditation: A Randomized Controlled Trial

**DOI:** 10.1155/2013/984678

**Published:** 2013-07-10

**Authors:** Carolina Baptista Menezes, Maria Clara de Paula Couto, Luciano G. Buratto, Fátima Erthal, Mirtes G. Pereira, Lisiane Bizarro

**Affiliations:** ^1^Faculdade de Psicologia, Universidade Federal de Pelotas, Avenida Duque de Caxias 250, 96030-001 Pelotas, RS, Brazil; ^2^Laboratório de Psicologia Experimental, Neurociências e Comportamento, Universidade Federal do Rio Grande do Sul, Ramiro Barcelos 2600, Sala 105, 90035-003 Porto Alegre, RS, Brazil; ^3^Universidade Federal do Rio Grande do Sul, Ramiro Barcelos 2600, Sala 104, 90035-003 Porto Alegre, RS, Brazil; ^4^Department of Psychology, Durham University (Queen's Campus), Stockton on Tees TS17 6BH, UK; ^5^Instituto de Biofísica Carlos Chagas Filho, Universidade Federal do Rio de Janeiro, Avenida Carlos Chagas Filho 373, Prédio do CCS, Bloco G, Sala G2-035, Rio de Janeiro, RJ, Brazil; ^6^Laboratório de Neurofisiologia do Comportamento, Universidade Federal Fluminense, Hernani Pires de Mello 101, São Domingos, 24.210-130 Niterói, RJ, Brazil

## Abstract

Self-regulatory trainings can be an effective complementary treatment for mental health disorders. We investigated the effects of a six-week-focused meditation training on emotion and attention regulation in undergraduates randomly allocated to a meditation, a relaxation, or a wait-list control group. Assessment comprised a discrimination task that investigates the relationship between attentional load and emotional processing and self-report measures. For emotion regulation, results showed greater reduction in emotional interference in the low attentional load condition in meditators, particularly compared to relaxation. Only meditators presented a significant association between amount of weekly practice and the reduction in emotion interference in the task and significantly reduced image ratings of negative valence and arousal, perceived anxiety and difficulty during the task, and state and trait-anxiety. For attention regulation, response bias during the task was analyzed through signal detection theory. After training, meditation and relaxation significantly reduced bias in the high attentional load condition. Importantly, there was a dose-response effect on general bias: the lowest in meditation, increasing linearly across relaxation and wait-list. Only meditators reduced omissions in a concentrated attention test. Focused meditation seems to be an effective training for emotion and attention regulation and an alternative for treatments in the mental health context.

## 1. Introduction

From a psychological point of view, meditation is a broad term that refers to a variety of techniques that aim to develop self-regulatory skills in the emotional and cognitive domains [1]. There has been an increased interest in the scientific study of meditation practices, in both its clinical application in the health care context and in the understanding of its mechanisms of action [2, 3].

From a theoretical and practical perspective, meditation seems to be particularly related to greater mental health [4, 5]. In psychological terms, one of the rationales underlying the meditation practice is that it comprises a form of mental training through which practitioners try to develop and increase flexibility and awareness of their mental processes, culminating in mental stability, well-being, and emotional balance [6]. Accordingly, results from randomized trials have demonstrated that meditation interventions as short as one or two months long can promote psychological improvements in patients with disorders such as social anxiety [7, 8], depression [9], and distress [10], as well as in healthy samples [11, 12].

It has been discussed that these outcomes likely reflect more adaptive emotion regulatory strategies, particularly better control of attention allocation, which allows the interruption of negative emotional processing [5, 13]. Noteworthy, attentional training constitutes one of the fundamental principles upon which meditation practices develop [3, 6], and it has been regarded as a cornerstone for any self-regulatory training from a cognitive perspective [14]. Thus, there seems to be an interesting parallel between psychological and meditation premises on how to foster emotion regulation.

Taking these considerations into account, the present study aimed to validate a secular focused meditation training by evaluating its effects on the ability to control attention allocation to negative stimuli in a healthy sample of undergraduates. The technique employed resembles *dharana* meditation [3], but, instead of using a mantra, it consisted of focusing attention on counting the out breath in order to avoid any direct link to a specific philosophy or doctrine in the university environment. The meditation group was compared to a relaxation and a wait-list control group, and the training schedule consisted of six weekly meetings.

Both emotion and attention regulations were assessed through a behavioral paradigm—the discriminative task [15]. The task comprises the random display of either a negative arousing or a neutral picture in the center of the screen, flanked by two peripheral bars. Participants are instructed to direct their eye gaze to the center while ignoring the task-irrelevant image and decide by a key press if the bars have the same orientation or not. There are two conditions, each requiring different levels of processing resources to succeed on the task. These are referred to as easy and difficult load conditions, in which subjects have to discriminate bars with a 90° or 6° difference in orientation, respectively. The emotion impact index is represented by the interference of the irrelevant negative image on the relevant attention task for each condition. According to the load theory [16], greater allocation of attentional resources to a relevant task may help reduce interference from irrelevant emotional stimuli. Thus, attentional deployment away from emotional stimuli, imposed by task constraints, may reduce emotional response and be an effective emotion regulation strategy [17, 18]. In the present study, we did not expect differences among groups for the difficult load condition, given that its exogenous attentional load was probably high enough to modulate emotion interference for all groups (see [15]). However, it was expected that in the easy condition only those trained in meditation would reduce emotion interference after training. Regardless of the low attentional load of the easy task, meditators were expected to have developed endogenous attentional control and greater attentional deployment away from the emotional stimuli [1]. Those trained only in relaxation or the wait-list controls were not expected to show such control.

Additionally, to further explore their attention regulation capacity, given that the task demanded an executive attention operation—paying attention to, discriminating, and deciding about the difference between two peripheral bars, we relied on signal detection theory (SDT) analyses [19]. In particular, the response bias index was used as it has been related to attentional control [20–22]. Response bias can be interpreted as the tendency to prioritize one of two answers, normally indicating that the subject adopts a strategy. In other words, it is a readiness to automatically give the same response [19, 23]. Given that attention training was an exclusive component of the meditation intervention and based on the importance of attention for self-regulation, we expected that only meditators would decrease response bias.

## 2. Methods

### 2.1. Participants

College students from the Universidade Federal do Rio Grande do Sul were invited to take part in the study through email and posters spread around its three campuses. Five hundred and twenty four students volunteered. After an online screening survey, participants were excluded if they were not in the range of 20–40 years old, did not have normal or corrected to normal vision, had any psychiatric or neurologic disorder, were taking any psychoactive medication, were undergoing psychotherapy treatment, and had had previous experience with meditation or yoga. One hundred students (57% female, 92% single, 81% with up to five minimum wage income, mean age 25 years, SD = 4.41) eligible for participation were randomly assigned to one of three groups: focused meditation (FM = 35), progressive relaxation (PR = 37), or wait-list control (WLC = 28). Seventy-four participants concluded the experiment (FM = 26, PR = 24, WLC = 24), of whom 41% were female, 70% single, 59% with up to five minimum wage income, and with a mean age of 25 years, SD = 4.44. None of these variables differed among groups (*P* ≥ .05), nor did attrition rates [*χ* ^2^(2) = 3.60, *P* = .16]. The Federal University of Rio Grande do Sul Ethics Committee (Institutional Review Board) approved this study. Participants provided written informed consent before the data were collected.

### 2.2. Design

This study comprised a randomized controlled trial. For the discrimination task, we employed a 2 (distractors' emotional load: neutral versus negative) × 2 (trial's attentional load: easy versus difficult) × 3 (group: focused meditation versus progressive relaxation versus wait-list control) design with repeated measures on the first two factors.

### 2.3. Assessment

#### 2.3.1. Screening

*Sociodemographic Questionnaire*. Created for the present study in order to investigate sociodemographic variables and exclusion criteria variables.

*Self-Report Questionnaire*—*SRQ [24].* The SRQ consists of 23 questions that investigate minor and psychotic psychiatric symptoms through yes/no answers. The validated Brazilian version, whose sensitivity and specificity coefficients are 83% and 80%, respectively, was used [25]. The cutoff point for female and male was 7 and 6 positive answers, respectively.

#### 2.3.2. Pretest and Posttest

*State and Trait Anxiety Inventory (STAI) [26].* The STAI comprises two scales measuring state and trait anxiety through twenty questions each. Answers are given on a 4-point Likert scale (1 = *not at all*, 4 = *very much*). The state and trait questions represent how the person feels at the present moment and normally, respectively. The higher the score, the greater the anxiety levels. The validated Brazilian version was used. Cronbach's alpha for the state scale is .89 and .88 for the trait [27].

*Concentrated Attention Test (Teste de Atenção Concentrada—AC*)* [28].* AC is a Brazilian psychometric test that assesses focused attention, with a test-retest coefficient of .73. For a maximum of 5 minutes, participants should mark only three types of triangles, among many others, all randomly distributed in rows on a paper sheet. Assessment of focused attentional performance includes correct answers, errors, omissions, and total score.

*Adult Self-Report Scale—ASRS [29].* The ASRS consists of 18 items, contemplating attention deficit and hyperactivity disorder (ADHD) symptoms adapted to adult life. Answers are given on a 5-point scale (0 = *never*, 1 = *rarely*, 2 = *sometimes*, 3 = *often*, and 4 = *very often*). Positive answers include “often” and “very often,” and for some questions (items 3, 4, 5, and 9 for part A and items 2, 7, and 9 for part B) “sometimes.” Cut-off point for possible diagnosis includes a minimum of 6 symptoms in at least one domain (inattention items 1–9 from part A and hyperactivity items 1−9 from part B), or both, and a score above 24 is considered highly suggestive of diagnosis. The ASRS was used in order to compare these symptoms across groups. If groups differed, this variable would be controlled for in AC and discrimination task analyses. 

**Figure 1:**
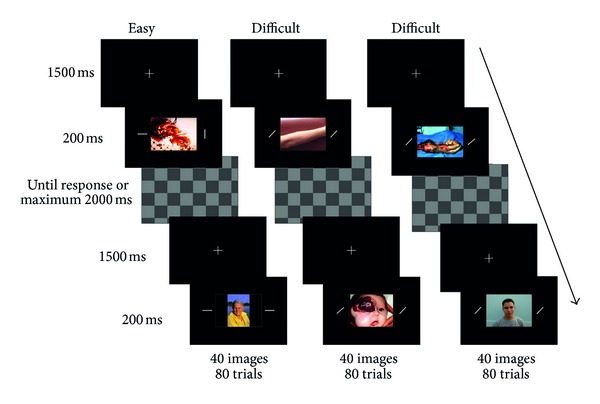
Experimental design: a centered fixation cross was presented for 1,500 ms, followed by a central picture and two peripheral bars, presented simultaneously (for 200 ms) to the right and left sides of fixation. Then a checkerboard-like mask was presented; this remained on the screen until the response was made or 2,000 ms had elapsed. Subjects were instructed to ignore the central picture and attend to the peripheral bars, responding with a keypress as quickly and accurately as possible whether the bars were in the same or a different orientation.

*Discrimination Task [15].* Figure 1 illustrates the trial structure. Each trial initiated with a fixation cross, shown for 1,500 ms. Next, a central picture (9° × 12°) and two peripheral bars (0.3° × 3.0°) were presented for 200 ms. The bars were at 9° to the right and left of the center of the picture. A whole-screen checkerboard mask was then shown, remaining on the screen until the subject responded or for 2,000 ms, which was the response deadline. The subjects were instructed to ignore the task-irrelevant central images and to respond as quickly and as accurately as possible whether or not the orientations of the peripheral bars were the same. Keypresses (with the right or left index finger) corresponding to same/different orientations (“q” or “p”) were counterbalanced across subjects. Two classes of images were employed: “neutral” (NE) and “emotional/unpleasant” (EM). Neutral images consisted of photographs of people, and unpleasant images consisted of photographs of mutilated bodies. We chose mutilated bodies because these are considered to be a very impacting category of emotional stimuli, likely to cause interference. Indeed, it has been already demonstrated that these images are efficient in generating an interference effect in the same paradigm used in the present study [15]. One hundred and twenty different images, 60 neutral and 60 unpleasant were utilized. A different set of pictures was used in the pretest and posttest sessions, and in each session pictures were repeated once. Forty-two images (14 neutral and 28 unpleasant) were taken from the International Affective Picture System (IAPS) developed by Lang and colleagues [30], and the remaining ones were obtained from the Internet. For the latter group of images, following the protocol developed by Lang and colleagues, all images were assessed on a 1–9 scale in terms of valence (from *negative* to *positive*) and arousal (from *low* to *high*) by a group of undergraduate students (*N* = 20, *M*_age_ = 22.3 years, SD = 1.8) using the paper-and-pencil version of the Self-Assessment Manikin [31]. Overall, images in the neutral category had mean valence ratings of 5.0 and mean arousal ratings of 3.3; images in the unpleasant category had mean valence ratings of 2.2 and mean arousal ratings of 6.4. The experimental session started with three training blocks containing 20 trials each, which were followed by three regular blocks of trials (80 trials each). The order of neutral and unpleasant images within a block was randomized. During training blocks, all images were photographs of objects, such as tools and furniture. During each experimental block, the difficulty of the bar-orientation task was fixed. One “easy” (EA) and two “difficult” (DF) blocks were obtained by manipulating the angular difference of the bars on nonmatch trials: 90° in easy blocks and 6° in the difficult blocks. There were two difficult blocks to guarantee the necessary number of correct answers in this condition. Each block contained the same number of match and nonmatch trials and the same number of neutral and unpleasant images. Valence and arousal levels for emotional and neutral images presented in each block type were matched to avoid differences in emotionality between blocks. During the training blocks, participants received feedback, which indicated anticipatory responses (reaction times—RT—less than 100 ms), slow responses (RT greater than 2,000 ms), and whether an incorrect key was pressed; during training, the RT was also indicated on the screen after each trial. Experimental blocks, which followed the training blocks, lasted approximately 5 min each, and their order was randomized across subjects. The subjects sat approximately 60 cm from the display, and the stimuli were presented with the software E-Prime.

*Task Ratings*. Two analog scales were used to assess how anxious participants felt during the task (anxiety DT) and how difficult they thought the task was (difficulty DT). Answers were given on a 10-point scale (0 = *not at all*, 10 = *very much*).

*Picture Ratings*. Participants viewed the pictures previously presented in the task in order to assess their valence and arousal. In total, 4 blocks were presented: 20 negative pictures from the easy condition, 20 neutral pictures from the easy condition, 20 negative pictures from the difficult condition, and 20 neutral pictures from the difficult condition. For the difficult condition, because there were two blocks during the behavioral task, the 20 negative and 20 neutral pictures were randomly selected from both blocks. The set of images in the behavioral task was different from pre- to posttest. Thus, the set of images for the ratings was also different for pre- and posttest. For the 4 blocks in the picture rating, images were displayed for 1 sec, and in the end participants had 15 sec to rate the block using the paper-and-pencil version of the Self-Assessment Manikin [31]. For both valence and arousal subjects rated the block from 1 (*very unpleasant and very relaxing, resp*.) to 9 (*very pleasant and very alerting, resp*.).

*Program Rating*. At the end of the 6-week training, participants rated the meditation and relaxation programs' quality (1 = *very bad*, 2 = *bad*, 3 = *indifferent*, 4 = *good*, and 5 = *very good*) and the usefulness of practices (1 = *not at all*, 2 = *a little*, and 3 = *very much*).

#### 2.3.3. During Intervention

*Practice Record*. Every week participants received and completed a form to register the frequency and duration of practice at home.

### 2.4. Procedure

After advertisement, volunteers interested in taking part in the study were sent the screening questionnaires online. Those eligible to participate were contacted to schedule a visit to the laboratory for the pretest session, which occurred during two weeks prior to the beginning of the trainings for all participants. Two assessments, one at pretest and another at posttest (before and after training, resp.), were carried out at the Laboratory of Experimental Psychology, Neuroscience, and Behavior, at the Institute of Psychology, Federal University of Rio Grande do Sul. The following sequence of assessment was used: STAI-S, AC, STAI-T, discrimination task, task ratings, ASRS, and picture ratings. The reason for determining this sequence was two-fold: to avoid the influence of the experimental task in the anxiety measures, as well as in the concentrated attention test, and to avoid leaving the task for the last assessment, as this could affect performance due to the amount of previous testing. Students were randomly assigned to one of the three groups. FM and PR trainings included 6 weekly meetings, each lasting one hour and thirty minutes. For each of them, there were four concurrent groups undergoing training at different times during the week. Posttest sessions also occurred during the two weeks after the training, following the same assessment sequence. WLC participants did not have any activity between testing sessions but did receive the meditation training after final testing. Training sessions were conducted by one of the authors, a psychologist with group experience, extensive training, and regular personal practice of yoga and meditation. Meetings were held in classrooms in the three campuses. Training sessions always started with a brief discussion about participants' weekly practice, difficulties, and experiences, followed by instructions, breathing exercises, formal practice—FM or PR—and again a brief discussion about the experience with that particular meeting. In the first and second meetings, formal practice lasted 15 and 20 minutes, respectively. For the following meetings, practices lasted 30 minutes. For the FM, participants could either sit cross-legged on a mat or on a chair with their feet on the ground. Because everyone was a beginner, they were instructed to pay attention to their own breathing, trying to slightly prolong the exhalation. Also, in order to characterize focused meditation, as well as to maintain their focus to this process and to the present moment, they were instructed to count their exhalation (mantras were not used in order to avoid any direct links to a specific philosophical or religious tradition). In the first half of the training, counting consisted of cycles fromof 1 to 10, and, for the next half, participants counted backwards from 100 to 1 (always one number per exhalation). PR sessions were formatted the same way, but all participants lay down on the mat for formal practice, which consisted of successive exercises of tension-relaxation for specific muscle groups [32]. A different muscle group was focused in each session (1st = wrists and arms; 2nd = face—forehead, eyes, nose, mouth, jaw; 3rd = neck; 4th = shoulders, chest, back, and abdomen; 5th = legs, feet; 6th = all together). Many repetitions of tension (±7 sec) and relaxation (±30 sec) were performed, after which people were guided to relax each part of the body, trying to keep alert during the whole process for the remaining time. For both groups, in the first meeting we provided a CD specially recorded for the study, with each guided practice, in order to help the daily training at home, as well as the practice record forms, which were collected in the last meeting or posttest session. 

### 2.5. Statistical Analyses

#### 2.5.1. Pretest

At pretest, a oneway ANOVA was performed to compare all self-report measures among groups and between gender.

For the discrimination task, all anticipatory and slow responses (<100 ms and >2,000 ms, resp.) were excluded from analyses; eliminated trials were infrequent at pre- and posttest (1.01% and 1% of the trials, resp.).

To explore the modulation produced by the emotional pictures we calculated the median reaction time (RT) and error rate (ER) for neutral and negative trials for each participant. These were the dependent variables, included in a factorial general linear model (GLM) for repeated measures considering load (easy versus difficult) and valence (emotional versus neutral) as within factors and group (FM versus PR versus WLC) as a between-subjects factor. We ran separate analyses for RT and ER.

A signal detection theory (SDT) analysis [19] was used to explore the effects of the meditation intervention over attentional control. First we calculated hits and false alarms, and response bias (*k*) analyses were conducted on the proportion of correct responses ((*k*) “Same” rate: the tendency to respond “same”, regardless of trial status) [19]. These were included in a GLM for repeated measures, with the same factors described above.

#### 2.5.2. Posttest

At posttest, the same GLMs were again carried out but with time as an additional within-subjects factor (pretest versus posttest). ANOVAs, polynomial contrasts, and pairwise comparisons using *t*-test were applied when appropriate. For all analyses, the SPSS 20.0 was used, and the alpha level for statistical significance was *P* = .05.

## 3. Results

### 3.1. Pretest

All measures were compared between drop-outs (i.e., participants who did not complete the study) and completers (i.e., participants who completed training and both testing sessions), and no significant differences were found. There were neither differences nor interactions between completers' groups in any of the variables analyzed. Of particular importance for this study, groups did not differ on attention deficit-hyperactivity disorder symptoms as measured by the ASRS (*F*(2,88) = 1.81,  *P* = .17, FM: *M* = 20.4, SD = 5.20, PR: *M* = 21.2, SD = 3.80; WLC: *M* = 22.00, SD = 3.40).

#### 3.1.1. Discrimination Task

There were no differences among groups for any of the task analyses (*P* > .05). A general emotional interference produced by the presence of a negative picture while subjects performed the discriminative task was revealed by the main effect for valence in the RT (*F*(1,94) = 11.59, *P* = .001). Participants were slower to perform the task when the central picture was negative (*M* = 600 ms, SD = 171) than neutral (*M* = 582 ms, SD = 148). The assumption that the difficulty of the bar orientation task was increased by reducing the angular difference between the bars was corroborated by a main effect of load in the ER outcome (*F*(1,94) = 803.3, *P* < .001; DF > EA). As expected, error rates were increased during the difficult condition (*M* = 45%, SD = 8.2) in comparison to the easy condition (*M* = 11%, SD = 10.9). 

Using signal detection theory analysis, results showed that in the difficult condition response bias was significantly greater (*F*(1, 96) = 117.6, *P* < .001; *M* = .73, SD = .18)) than in the easy condition (*M* = .50, SD = .04).

#### 3.1.2. Self-Report Assessments

There were no differences among groups (*P* > .05). Results are presented in Table 1.

### 3.2. Posttest

#### 3.2.1. Practice Record and Program Ratings

Weekly practice for the FM group varied between 2 and 5 times (*M* = 3.01, SD = 1.06) and 17–115 minutes per week (*M* = 50 min., SD = 26.9). For PR, practice varied between 2 and 4 times (*M* = 3.03, SD = .94) and 17–76 minutes (*M* = 48 min., SD = 16.00). There were no significant group differences for these variables (*P* = .96, and *P* = .89, resp.). Program ratings did not differ between FM (quality: *M* = 4.60,  SD = .49; usefulness: *M* = 2.80,  SD = .42) and PR (quality: *M* = 4.50, SD = .50; usefulness: *M* = 2.80, SD = .36) (quality: *t*(48) = .45, *P* = .65; usefulness: *t*(46) = −.64, *P* = .52) groups.

#### 3.2.2. Discrimination Task

The reaction time analysis of the emotional interference effect produced by viewing emotional pictures revealed an interaction between valence and time (*F*(1, 66) = 4.1, *P* = .045). Performing the discrimination task in the presence of an emotional picture was significantly different from neutral picture only at pretest (*t*(96) = 3.40, *P* = .001), but not at posttest (*t*(70) = 1.7, *P* = .09). 

**Figure 2:**
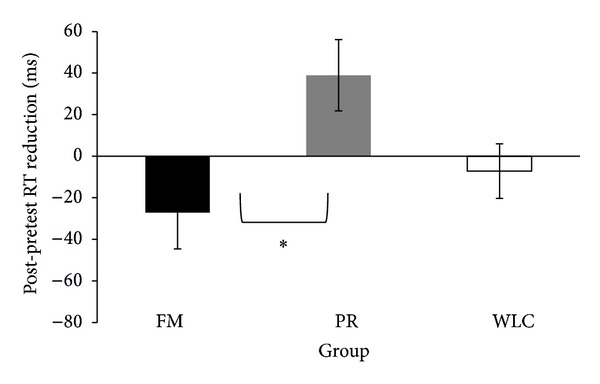
Mean values (ms) representing the subtraction of posttest reaction times from pretest reaction times for emotional images in the easy condition. Negative values indicate a reduction of picture interference in the task. Standard errors are represented by the error bars. FM: focused meditation; PR: progressive relaxation; WLC: wait-list control. A one-way ANOVA indicated a significant difference among groups, and pairwise comparisons revealed that the meditation group presented a significantly greater reduction than the relaxation group. *P < .05.

According to our hypothesis, we expected a reduction of the emotional effect of negative stimuli in the easy condition in the posttest session for the FM group. To test this difference, we created a variable to represent modulations in reaction time due to intervention. The variable was calculated by subtracting the reaction times in the posttest condition from the reaction times in the pretest condition for emotional and neutral images, each separately, and for each load condition (easy and difficult). Thus, there were a variable representing the subtraction in reaction time for the emotional images in the easy and in the difficult condition and a variable representing this subtraction for neutral images in the easy and the difficult condition. Negative values would indicate a reduction of picture interference on the main discrimination task. After performing a GLM for this variable for each load condition separately, results partially corroborated our hypothesis. We analyzed data from each load condition separately because, as discussed previously, they represent conditions with very different levels of neural resources available to process the distractive pictures and group effect that were not expected to be present in the difficult load condition. We found a trend towards an interaction between valence and group in the easy condition (*F*(2,66) = 2.38, *P* = .10) but not in the difficult condition (*F*(2,66) = 1.71, *P* = .20). To further explore these results, we carried out planned comparisons for emotional and neutral images in the easy condition. Meditators presented a significantly greater reduction of emotion interference in comparison to the relaxation group (*t*(46) = 2.69, *P* = 0.01) but not in comparison to the wait-list control group (*t*(43) = −.88, *P* = .37) (Figure 2). There were no significant differences between groups for neutral images (all *P*s > .05).

In order to make sure that this result indicated a reduction in the emotional interference by negative stimuli among meditators and not just a tradeoff between speed and error, we also created the same index subtracting ER for emotion images from pre- to posttest, and there were no group differences (*F*(2,66) = 1.47, *P* = .23).

**Figure 3:**
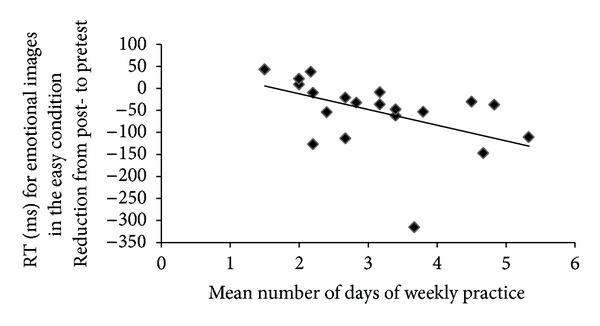
Association between the mean number of days of weekly practice and the variable representing the post-pretest reduction in RT (ms) for emotional images in the easy condition for the FM group. Number of days of practice was not reported by six participants.

Finally, to explore if the amount of meditation practice could predict the ability to reduce the emotional impact of negative stimuli in the easy condition, we conducted a linear regression analysis between mean days of weekly practice and the index of reduction of picture interference described above. Only in the FM group there was a negative relationship between number of days of weekly practice and the variable representing the reduction in RT for emotional images (*r* = −.40, *P* = .04) (Figure 3).

Considering the ER as the outcome, we found only a main effect for load (*F*(1, 66) = 689.4, *P* < .001; DF > EA).

**Figure 4:**
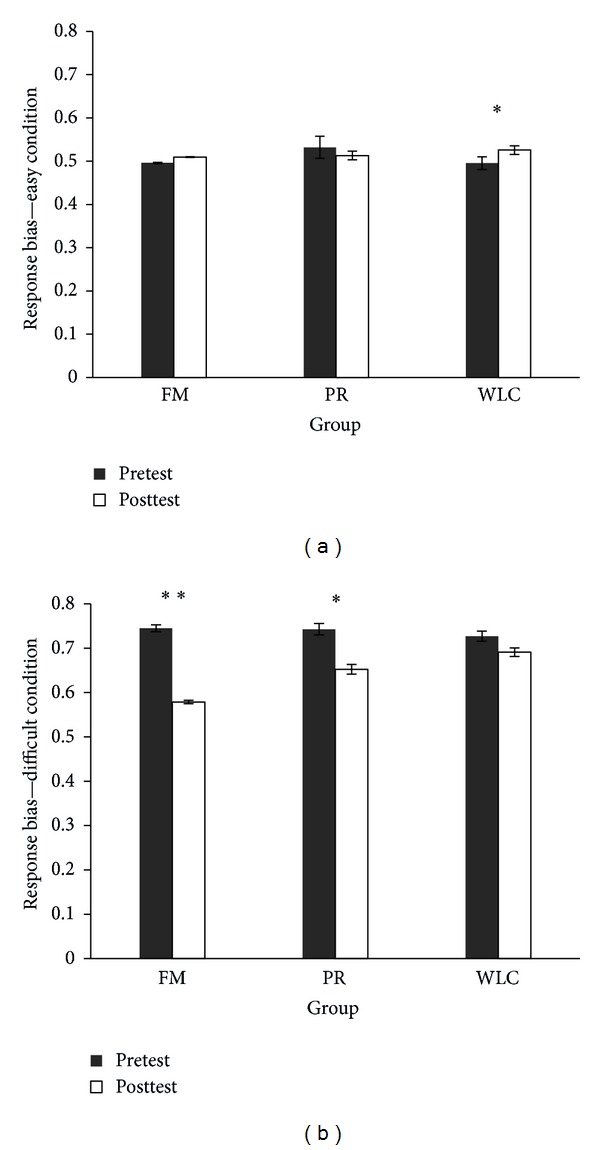
Mean response bias rate at pre- and posttest for each group. FM: focused attention meditation; PR: progressive relaxation; WLC: wait-list control. Standard errors are represented by the error bars. (a) In the easy condition, there was a significant increase in response bias at posttest for the wait-list control group. *P < .05. (b) In the difficult condition, participants from both meditation and relaxation groups significantly reduced response bias at posttest. **P < .001; *P < .01, respectively.

For SDT analysis, response bias remained higher in the difficult condition (*F*(1, 68) = 81.1, *P* < .001; DF > EA) but was significantly reduced at posttest (*F*(1, 68) = 23.1,  *P* < .001; pretest > posttest). Importantly, there was a significant three-way interaction (load × time × group: *F*(2, 68) = 4.0, *P* = .02). More specifically, in the easy condition at posttest, there was an increase in response bias only in the WLC (FM: *t*(23) = −1.3, *P* = .18; PR: *t*(23) = 1.0, *P* = .28; WLC: *t*(22) = −2.7, *P* = .01), and in the difficult condition response bias was significantly reduced in the FM and PR groups (FM: *t*(23) = 5.6, *P* < .001; PR: *t*(23) = 3.6, *P* = .001; WLC: *t*(22) = 1.0, *P* = .29) (Figure 4).

**Figure 5:**
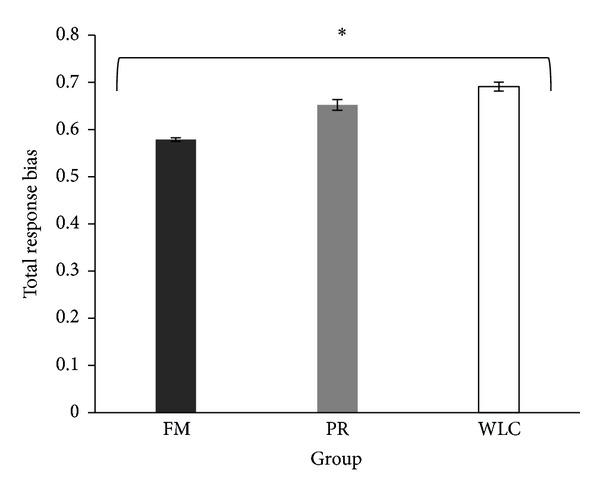
Total mean response bias rate for each group at posttest. FM: focused attention meditation; PR: progressive relaxation; WLC: wait-list control. Standard errors are represented by the error bars. There was a linear significant effect (*P < .05), in which the meditation group presented the smallest bias, followed by the relaxation group, and next by the wait-list control group.

Also, response bias followed a dose-response pattern at posttest, in which meditation practitioners presented the smallest bias, followed by relaxation and wait-list control (*F*(2, 68) = 4.0, linear test *P* = .02) (Figure 5).

#### 3.2.3. Self-Report Assessments

Table 1 shows results for self-report measures. After training, FM showed improvements in trait anxiety and some task ratings. Despite no significant interaction between group and time for concentrated attention parameters, paired comparisons revealed a significant reduction of omission errors only for meditation (FM: *t*(25) = 2.17, *P* = .03; PR: *t*(23) = 1.05, *P* = .30; WLC: *t*(21) = .53, *P* = .59). There were no significant correlations between amount of practice and any of the self-report measures for any of the active groups.

## 4. Discussion

The present study evaluated the effect of a six-week focused meditation training on emotion and attention regulation in a healthy sample of undergraduates, in comparison to a relaxation and a wait-list control group. Findings indicated that meditators presented greater reduction of emotion interference in the easy condition, which was not explained by a tradeoff with error rate, and which was complemented by a significant reduction in the subjective evaluation of negative valence and arousal of emotional images. Additionally, only meditators presented a significant reduction in state and trait anxiety and an increase in concentrated attention. Most importantly, the frequency of meditation practice predicted the reduction of interference produced by negative stimuli as revealed by a negative relationship between the number of days of weekly practice and the reduction in RT for emotional images. Finally, meditators presented a greater reduction in response bias in the difficult condition, which followed a dose-response pattern.

### 4.1. Meditation and Emotion Regulation

Behavioral studies show that experimental manipulation of attention reduces emotion interference produced by distractive emotional stimuli, especially when attention load to the main task is high [15, 33]. This idea gives support to the present findings, in which meditators presented the most pronounced reduction in emotional interference after training and group differences were present only in the easy condition. The difficult condition consisted of a high exogenous attentional load task [34], facilitating attentional deployment away from the emotional stimuli. In this condition, task load was so high that it may have exhausted processing resources and reduced the processing of the distractive emotional stimuli for all groups. However, in the easy condition, the task's exogenous demands were lower, freeing up participants' resources to process the distractive pictures. In this condition, it was expected that emotional pictures would produce an interference on behavior, revealed by increased reaction times. The results showed that meditators were better to regulate interference from emotional pictures. Reaction times when negative stimuli were presented were reduced after meditation training. This indicates that meditators were able to control their attention better to perform the bar discrimination task, reducing the interference of emotional distractive information. Their increased ability to control attention allowed them more successfully to deploy attention as an emotional regulation strategy [17, 18].

One limitation, however, was the lack of a memory test for the images after the task. A better recall of emotional images might have indicated a more efficient use of divided attention, instead of better selective attention. Nevertheless, we believe this is not the case, given that meditators produced less negative valence ratings and lower arousal ratings. Also, they specifically practiced focused meditation, in which selective and sustained attentions are trained in order to inhibit distractions—internal or external—or disengage faster from them [1]. Studies investigating the efficacy of different emotion regulation strategies have demonstrated that selective attention allocation helps reducing emotional reactivity [18]. In fact, attention allocation may be more effective than other strategies, such as reappraisal and suppression [35–37], and one possible explanation relates to their temporal distinction, given that attention allocation takes place faster, impacting earlier stages of emotion-generative processes [38].

In line with these assumptions, other studies have also observed the efficacy of meditation for emotion regulation [39–41] and that psychological improvements following meditation training were mediated by enhanced top-down control [12, 42]. Interestingly, meditation can be more effective than distraction—an attention-allocation strategy—in reducing reactivity to negative self-beliefs related to social anxiety disorder [7]. One hypothesis for this outcome is the idea that meditation comprises a combination of an attentive mind with an emotional state of relaxation [3, 43, 44].

Our results showed that only meditators had a significant reduction in trait and state anxiety. This finding is particularly relevant, given that higher levels of anxiety can impair the regulatory process, biasing attention towards negative stimuli [45], or disrupting modulation of negative emotion [46]. Thus, cultivating attention stability along with a relaxation state seems to facilitate regulatory processes, possibly explaining why meditation may be distinct from other strategies, such as distraction [7] or relaxation [40].

### 4.2. Meditation and Attention Regulation

As stated previously, response bias can be interpreted as the tendency to prioritize one of two answers, normally indicating that the subject adopts a strategy. In other words, it is a readiness to respond the same thing in an automated fashion [19, 23]. In the context of repeated two-option forced choices, like in our task, people present less persistence in doing subsequent cognitive tasks, either solvable or unsolvable, indicating that resource depletion is related to impaired executive control [47]. Thus, the fact that in the present study response bias was significantly higher in the difficult condition across groups at pretest, but significantly lower at posttest, particularly in the meditation group, suggests an improvement in their executive control.

This is in line with other studies that have used different selective attention manipulations to investigate response bias [20–22]. For instance, a three-week attention training aiming to help subjects ignore distractors and process the target more efficiently in an auditory selection task produced a significant reduction in response bias after training, which correlated with neural response as indexed by P3 amplitude [21]. In other words, the more attention subjects allocated to the target, the more controlled and less automated were their responses. Likewise, it has been shown that reversing a pattern of response bias was only possible through selective attention training but not through training in which the manipulation involved a high load cognitive operation [22].

Greater executive control has been demonstrated in people who have participated in meditation training programs, as well as in experienced meditators [48–50]. Similarly, areas typically involved in executive control, such as lateral pre-frontal cortex and anterior cingulate cortex [51, 52], are more activated during meditation or during the execution of an attentional task by meditators when compared to controls [53–56].

It should be noted that one study which also used a discrimination task to investigate meditation effects on vigilance found no difference for response bias after a three-month meditation training [57]. However, in our results, response bias interacted with difficulty, and the task used by MacLean et al. did not include distractors nor had a condition that was more difficult than the other. Thus, in their study there might have been no reason for participants to adopt a strategy in the first place. In addition, participants were already meditators at pretest [57].

Finally, we highlight that this reduction is likely to have been intentional, goal-oriented, and not simply an inverse strategy or random response, because in the latter situations the result would have been accompanied by a higher error rate, which was not the case. Thus, in the difficult condition at posttest, meditators seem to have had more executive control over their goal-oriented behavior, which is in line with the hypothesis of greater attention efficiency in attention tasks among meditators [50, 58]. This regulation can also be inferred from the finding that only meditators significantly reduced omissions in the concentrated attention test, corroborating studies that used similar [3] as well as different measures of concentrated attention [59]. Importantly, given that a motivational reward, such as money, can facilitate attentional and conflict resolution performance [60], it is worth highlighting that our participants were not paid for their participation and that a potential interaction with this external motivational factor is ruled out in the present study.

### 4.3. Meditation as a Psychological Rehabilitation

Our results corroborate the idea that emotion and attention regulation are intertwined and that meditation can enhance these skills. Moreover, meditation seems to constitute a particular type of emotion regulation strategy, which can be clinically relevant. It is known that in some psychiatric patients, even in remission, such as remitted depressed patients, there is a difficulty in reducing amygdala's reactivity to negative emotional stimuli when using reappraisal, and this correlates with the report of significantly less use of such strategy on a daily basis [61]. Likewise, there is evidence showing that anxiety patients present a bias favoring amygdala overactivation, as well as under-recruitment of prefrontal areas in the processing of negative stimuli [45].

Thus, self-regulation practices, such as the meditation training proposed in the present work, seem to be an alternative for clinical conditions, especially considering the early effects of attention on emotion response [38], and the fact that early reactivity to emotional stimuli may modulate subsequent processing stages [62]. Accordingly, Farb et al. [13] have recently discussed that among patients with affective disorders, mastering the direction of attention can help limiting the cognitive elaboration of negative emotions and negative self-evaluation.

These assumptions are in accordance with studies that have demonstrated a positive effect of meditation training in psychiatric symptoms and disorders [5, 7, 63, 64]. For instance, a meta-analysis showed that in patients with anxiety and mood disorders the effect sizes of meditation-based interventions were very robust, independent of year of publication, and maintained over follow-up [65]. It should be noted that in addition to its therapeutic effects, meditation could also contribute to mental health practices by fostering therapist's effectiveness, therapeutic alliance, and complementary perspectives on therapeutic processes [4]. 

## Figures and Tables

**Table 1:** General linear model for repeated measures: changes in self-report assessments from Pre- to posttest for each group.

Questionnaires	GLM				Paired *t*-tests			
			FM		PR		WLC	
			Pretest	Posttest	Pretest	Posttest	Pretest	Posttest
	df	*F*	*M* (SD)	*M* (SD)	*M* (SD)	*M* (SD)	*M* (SD)	*M* (SD)
Anxiety								
STAI state	2.69	9.34*	1.93 (.41)	1.78 (.39)	1.92 (.37)	1.78 (.29)	1.78 (.35)	2.16 (.49)*
STAI trait	2.69	9.75*	1.94 (.44)	1.78 (.33)*	1.95 (.43)	1.83 (.43)	1.90 (.34)	2.16 (.42)*
Attention								
AC-correct answer	2.69	.19	105.7 (20.8)	118.5 (19.4)	101.6 (24.8)	111.3 (23.4)	105.8 (18.8)	117.7 (17.7)
AC-errors	2.69	.58	.53 (.90)	1.19 (4.6)	1.04 (1.9)	.75 (1.6)	.59 (.79)	.41 (.73)
AC-omissions^a^	2.69	.49	10.8 (8.3)	7.1 (5.2)*	10.7 (14.1)	8.4 (7.1)	13.1 (7.8)	12.2 (5.6)
AC-total score	2.68	.35	93.6 (21.9)	111.0 (20.3)	89.8 (29.8)	102.2 (24.7)	92.5 (20.5)	107.2 (20.5)
Task ratings								
Anxiety DT	2.53	4.50*	5.73 (2.62)	3.95 (2.0)*	5.09 (2.2)	5.24 (2.1)	5.81 (2.5)	6.50 (2.0)
Difficulty DT	2.53	5.0*	6.94 (2.0)	5.26 (1.9)**	6.90 (1.8)	6.90 (1.8)	7.25 (2.1)	7.38 (1.3)
VAL-E	2.68	3.86*	1.43 (.47)	1.70 (.55)*	1.44 (.69)	1.70 (.73)	1.81 (.82)	1.47 (.64)
ARO-E	2.68	3.41*	7.75 (1.2)	6.97 (.90)*	7.24 (1.8)	7.40 (.97)	7.36 (1.5)	7.47 (1.3)
VAL-N	2.68	.31	5.68 (.85)	6.18 (1.3)	5.98 (1.3)	6.24 (1.3)	6.18 (.99)	6.43 (1.3)
ARO-N	2.68	.09	2.81 (1.5)	2.77 (1.5)	3.24 (1.8)	3.22 (1.5)	2.47 (1.5)	2.61 (1.3)

GLM: general linear model for repeated measures; FM: focused meditation; PR: progressive relaxation; WLC: wait-list control; STAI: state trait anxiety inventory; AC: atenção concentrada (concentrated attention); DT: discriminant task; VAL-E: assessment of valence in emotional condition; ARO-E: assessment of arousal in emotional condition; VAL-N: assessment of valence in neutral condition; ARO-N: assessment of arousal in neutral condition.

^a^Student's *t*-test.

*P < .05; **P < .001.

## References

[1] Lutz A, Slagter HA, Dunne JD, Davidson RJ (2008). Attention regulation and monitoring in meditation. *Trends in Cognitive Sciences*.

[2] Lutz A, Dunne JD, Davidson RJ, Zelazo P, Moscovitch M, Thompson E (2007). Meditation and the neuroscience of consciousness: an introduction. *Cambridge Handbook of Consciousness*.

[3] Telles S, Raghavendra BR (2011). Neurophysiological changes in meditation correlated with descriptions from the ancient texts. *Biofeedback*.

[4] Abbey SE (2012). Mindfulness and psychiatry. *The Canadian Journal of Psychiatry*.

[5] Chambers R, Gullone E, Allen NB (2009). Mindful emotion regulation: an integrative review. *Clinical Psychology Review*.

[6] Wallace BA, Shapiro SL (2006). Mental balance and well-being: building bridges between Buddhism and Western psychology. *The American Psychologist*.

[7] Goldin PR, Gross JJ (2010). Effects of Mindfulness-Based Stress Reduction (MBSR) on emotion regulation in social anxiety disorder. *Emotion*.

[8] Goldin P, Ziv M, Jazaieri H, Hahn K, Gross JJ MBSR Vs. aerobic exercise in social anxiety: fMRI of emotion regulation of negative self-beliefs. *Social and Cognitive Affective Neuroscience*.

[9] Teasdale JD, Moore RG, Hayhurst H, Pope M, Williams S, Segal ZV (2002). Metacognitive awareness and prevention of relapse in depression: empirical evidence. *Journal of Consulting and Clinical Psychology*.

[10] Jain S, Shapiro SL, Swanick S (2007). A randomized controlled trial of mindfulness meditation versus relaxation training: effects on distress, positive states of mind, rumination, and distraction. *Annals of Behavioral Medicine*.

[11] Jacobs TL, Epel ES, Lin J (2011). Intensive meditation training, immune cell telomerase activity, and psychological mediators. *Psychoneuroendocrinology*.

[12] Sahdra BK, MacLean KA, Ferrer E (2011). Enhanced response inhibition during intensive meditation training predicts improvements in self-reported adaptive socioemotional functioning. *Emotion*.

[13] Farb NAS, Anderson AK, Segal ZV (2012). The mindful brain and emotion regulation in mood disorders. *The Canadian Journal of Psychiatry*.

[14] Wadlinger HA, Isaacowitz DM (2011). Fixing our focus: training attention to regulate emotion. *Personality and Social Psychology Review*.

[15] Erthal FS, de Oliveira L, Mocaiber I (2005). Load-dependent modulation of affective picture processing. *Cognitive, Affective and Behavioral Neuroscience*.

[16] Lavie N (2005). Distracted and confused?: selective attention under load. *Trends in Cognitive Sciences*.

[17] Mocaiber I, Pereira MG, Erthal FS (2010). Fact or fiction? An event-related potential study of implicit emotion regulation. *Neuroscience Letters*.

[18] Ochsner KN, Gross JJ (2005). The cognitive control of emotion. *Trends in Cognitive Sciences*.

[19] Macmillan NA, Creelman CD (2005). *Detection Theory: A User’s Guide*.

[20] Abrams J, Barbot A, Carrasco M (2010). Voluntary attention increases perceived spatial frequency. *Attention, Perception & Psychophysics*.

[21] Melara RD, Tong Y, Rao A (2012). Control of working memory: effects of attention training on target recognition and distractor salience in an auditory selection task. *Brain Research*.

[22] Miles JD, Proctor RW (2010). Attention is required for acquisition but not expression of new response biases. *Journal of Experimental Psychology*.

[23] Voss A, Rothermund K, Brandtstädter J (2008). Interpreting ambiguous stimuli: separating perceptual and judgmental biases. *Journal of Experimental Social Psychology*.

[24] Harding TW, de Arango MV, Baltazar J (1980). Mental disorders in primary health care: a study of their frequency and diagnosis in four developing countries. *Psychological Medicine*.

[25] de Jesus Mari J, Williams P (1986). A validity study of a psychiatric screening questionnaire (SRQ-20) in primary care in the city of Sao Paulo. *The British Journal of Psychiatry*.

[26] Spielberg CD, Gorsuch RL, Lushene RE, Biaggio AMB, Natalício L (1979). *Inventário de ansiedade Traço-Estado—IDATE*.

[27] Fioravanti ACM, Santos LF, Maissonette S, Cruz APM, Landeira-Fernandez J (2006). Avaliação da estrutura fatorial da Escala de Ansiedade-Traço do IDATE. *Avaliação Psicológica*.

[28] Cambraia SV (2003). *Teste AC—Manual*.

[29] Mattos P, Segenreich D, Saboya E, Louzã M, Dias G, Romano M (2006). Adaptação transcultural para o português da escala Adult Self-Report Scale para avaliação do transtorno de déficit de atenção e hiperatividade (TDAH) em adultos. *Revista De Psiquiatria Clínica*.

[30] Lang PJ, Bradley MM, Cuthbert BN (2005). *International Affective Picture System (IAPS): Affective Ratings of Pictures and Instruction Manual*.

[31] Bradley MM (1994). Measuring emotion: the self-assessment manikin and the semantic differential. *Journal of Behavior Therapy and Experimental Psychiatry*.

[32] Nieves MV, Vila J, Caballo VE (2002). Técnicas de Relaxamento. *Manual de Técnicas de Terapia e Modificação do Comportamento*.

[33] Morawetz C, Baudewig J, Treue S, Dechent P (2010). Diverting attention suppresses human amygdala responses to faces. *Frontiers in Human Neuroscience*.

[34] Chun MM, Golomb JD, Turk-Browne NB (2011). A Taxonomy of external and internal attention. *Annual Review of Psychology*.

[35] Kanske P, Heissler J, Schönfelder S, Bongers A, Wessa M (2011). How to regulate emotion? Neural networks for reappraisal and distraction. *Cerebral Cortex*.

[36] McRae K, Hughes B, Chopra S, Gabrieli JDE, Gross JJ, Ochsner KN (2010). The neural bases of distraction and reappraisal. *Journal of Cognitive Neuroscience*.

[37] Sheppes G, Meiran N (2007). Better late than never? on the dynamics of online regulation of sadness using distraction and cognitive reappraisal. *Personality and Social Psychology Bulletin*.

[38] Thiruchselvam R, Blechert J, Sheppes G, Rydstrom A, Gross JJ (2011). The temporal dynamics of emotion regulation: an EEG study of distraction and reappraisal. *Biological Psychology*.

[39] Farb NAS, Anderson AK, Mayberg H, Bean J, McKeon D, Segal ZV (2010). Minding one’s emotions: mindfulness training alters the neural expression of sadness. *Emotion*.

[40] Ortner CNM, Kilner SJ, Zelazo PD (2007). Mindfulness meditation and reduced emotional interference on a cognitive task. *Motivation and Emotion*.

[41] Taylor VA, Grant J, Daneault V (2011). Impact of mindfulness on the neural responses to emotional pictures in experienced and beginner meditators. *NeuroImage*.

[42] Jha AP, Stanley EA, Kiyonaga A, Wong L, Gelfand L (2010). Examining the protective effects of mindfulness training on working memory capacity and affective experience. *Emotion*.

[43] Kubota Y, Sato W, Toichi M (2001). Frontal midline theta rhythm is correlated with cardiac autonomic activities during the performance of an attention demanding meditation procedure. *Cognitive Brain Research*.

[44] Tang Y-Y, Posner MI (2009). Attention training and attention state training. *Trends in Cognitive Sciences*.

[45] Bishop SJ (2007). Neurocognitive mechanisms of anxiety: an integrative account. *Trends in Cognitive Sciences*.

[46] Mocaiber I, Pereira MG, Erthal FS, Figueira I, Machado-Pinheiro V, Cagy M (2009). Regulation of negative emotions in high trait anxious individuals: an ERP study. *Psychology & Neuroscience*.

[47] Vohs KD, Baumeister RF, Schmeichel BJ, Twenge JM, Nelson NM, Tice DM (2008). Making choices impairs subsequent self-control: a limited-resource account of decision making, self-regulation, and active initiative. *Journal of Personality and Social Psychology*.

[48] Chan D, Woollacott M (2007). Effects of level of meditation experience on attentional focus: is the efficiency of executive or orientation networks improved?. *Journal of Alternative and Complementary Medicine*.

[49] Froeliger B, Garland EL, McClernon FJ (2012). Yoga meditation practitioners exhibit greater gray matter volume and fewer reported cognitive failures: results of a preliminary voxel-based morphometric analysis. *Evidence-Based Complementary and Alternative Medicine*.

[50] Kozasa EH, Sato JR, Lacerda SS (2012). Meditation training increases brain efficiency in an attention task. *NeuroImage*.

[51] Miller EK, Cohen JD (2001). An integrative theory of prefrontal cortex function. *Annual Review of Neuroscience*.

[52] Posner MI, Fan J, Pomerantz JR, Crair MC (2004). Attention as an organ system. *Topics in Integrative Neuroscience: From Cells To Cognition*.

[53] Short EB, Kose S, Mu Q (2010). Regional brain activation during meditation shows time and practice effects: an exploratory FMRI study. *Evidence-Based Complementary and Alternative Medicine*.

[54] Brefczynski-Lewis JA, Lutz A, Schaefer HS, Levinson DB, Davidson RJ (2007). Neural correlates of attentional expertise in long-term meditation practitioners. *Proceedings of the National Academy of Sciences of the United States of America*.

[55] Dickenson J, Berkman ET, Arch J, Lieberman MD (2013). Neural correlates of focused attention during a brief mindfulness induction. *Social Cognitive and Affective Neuroscience*.

[56] Farb NAS, Segal ZV, Mayberg H (2007). Attending to the present: mindfulness meditation reveals distinct neural modes of self-reference. *Social Cognitive and Affective Neuroscience*.

[57] MacLean KA, Ferrer E, Aichele SR (2010). Intensive meditation training improves perceptual discrimination and sustained attention. *Psychological Science*.

[58] Slagter HA, Lutz A, Greischar LL, Francis AD, Nieuwenhuis S, Davis JM (2007). Mental training affects distribution of limited brain resources. *Plos Biology*.

[59] Lutz A, Slagter HA, Rawlings NB, Francis AD, Greischar LL, Davidson RJ (2009). Mental training enhances attentional stability: neural and behavioral evidence. *Journal of Neuroscience*.

[60] Padmala S, Pessoa L (2011). Reward reduces conflict by enhancing attentional control and biasing visual cortical processing. *Journal of Cognitive Neuroscience*.

[61] Kanske P, Heissler J, Schönfelder S, Wessa M (2012). Neural correlates of emotion regulation deficits in remitted depression: the influence of regulation strategy, habitual regulation use, and emotional valence. *NeuroImage*.

[62] Moratti S, Keil A, Stolarova M (2004). Motivated attention in emotional picture processing is reflected by activity modulation in cortical attention networks. *NeuroImage*.

[63] Chiesa A, Serretti A (2010). A systematic review of neurobiological and clinical features of mindfulness meditations. *Psychological Medicine*.

[64] Kocovski NL, Segal ZV, Battista SR (2009). Mindfulness and psychopathology: problem formulation. *Clinical Handbook of Mindfulness*.

[65] Hofmann SG, Sawyer AT, Witt AA, Oh D (2010). The effect of mindfulness-based therapy on anxiety and depression: a meta-analytic review. *Journal of Consulting and Clinical Psychology*.

